# Sexual and urological reconstruction following penectomy for penile cancer: phalloplasty

**DOI:** 10.1038/s41443-025-01161-z

**Published:** 2025-09-16

**Authors:** Andrea Gobbo, Andrew Nim Christopher, Angelo di Giovanni, Abdullah Al-Mitwalli, Karl Pang, David Ralph, Wai Gin Lee

**Affiliations:** 1 Department of Urology, University College London Hospital, 235 Euston Road, NW1 2BU London, United Kingdom; 2 Department of Biomedical Sciences, Humanitas University, Via Rita Levi Montalcini 4, Pieve Emanuele, 20072 Milan, Italy; 3 St Peter’s Andrology Centre and the Institute of Urology, University College Hospital, 235 Euston Road, NW1 2BU London, United Kingdom; 4 Division of Surgery and Interventional Sciences, University College London, Gower Street, WC1E 6BT London, United Kingdom

**Keywords:** Sexual dysfunction, Urogenital reproductive disorders

## Abstract

Phalloplasty following penectomy for penile cancer presents a complex reconstructive challenge, requiring restoration of urinary and sexual function while addressing anatomical and psychological sequelae. Unlike gender-affirming phalloplasty, this procedure is complicated by previous surgery and potential anatomical deficits. However, limited data exist to guide reconstruction in this cohort. This narrative review summarises available evidence on phalloplasty post-penectomy. A systematic search identified six relevant studies, including 48 patients, with follow-up ranging from 1 to 150 months. The radial artery free flap (RAP) and anterolateral thigh flap (ALTP) are the preferred techniques, each with distinct advantages and limitations. While RAP offers superior tactile recovery, ALTP reduces donor site morbidity. Complication rates are high, particularly urethral strictures and fistulae, affecting up to 64.3% of cases. Despite these risks, functional outcomes, including standing micturition and sexual activity, are achievable and patient satisfaction remains high. Challenges include extrapolating data from transgender cohorts and managing psychosocial concerns. A multidisciplinary approach is essential for optimising patient selection, counselling, and long-term outcomes. Further research is needed to refine surgical techniques, improve complication management, and explore innovative reconstructive strategies.

## Introduction

Phalloplasty following penectomy for penile cancer represents a significant challenge in reconstructive urology. This multifaceted procedure aims to reconstruct a functional and aesthetic neophallus to enable standing micturition, sexual function, and improve overall quality of life. In addition to other forms of genital reconstruction, this approach must address the potentially morbid anatomical, physiological, and psychological sequelae of penile cancer surgery [1–4].

Penile cancer, although rare, may necessitate partial or total penectomy for complete excision [5]. Such radical surgery may compromise urinary and sexual function, and body image [6]. Phalloplasty in this cohort of men is therefore complicated by surgical scarring and the potential loss of vascular structures [7]. There are limited published data specific to this population to guide reconstruction because most of the literature on phalloplasty focuses on transgender patients undergoing gender-affirming surgery [8, 9]. Extrapolating these findings to cisgender males post-penectomy may not be entirely appropriate.

Over the previous decades, the techniques for phalloplasty have evolved from loco-regional tissue transfer using pedicled abdominal flaps to more advanced microsurgical free flaps [10–12]. Contemporary techniques, including radial artery free flap phalloplasty (RAP) and anterolateral thigh flap phalloplasty (ALTP) result in a functional and aesthetic neophallus but with associated limitations and risks [13, 14]. Despite the technical progress, significant gaps remain in understanding long-term outcomes, optimal patient selection, and strategies to minimise complications [8]. This narrative review summarises the existing literature on phalloplasty following penectomy for penile cancer supplemented by expert insights from a high-volume centre.

## Methods

This narrative review was conducted to evaluate surgical techniques and outcomes of phalloplasty following penectomy for penile cancer. When possible, data were extracted from studies that included a mixed cohort of patients.

### Search strategy and selection criteria

A comprehensive search of MEDLINE and Embase databases was performed for English-language studies published from January 1946 to December 2024. The search terms included combinations of “penile cancer” AND “sex reassignment surgery” OR “reconstructive procedures” OR “surgical flaps” OR “phalloplasty”. The terms “penile cancer” AND “phalloplasty” were used as truncated keyword searches to improve inclusivity. Articles were screened for relevance based on title, abstract, and full text.

Inclusion criteria were:

- Case reports, case series, retrospective, or prospective studies detailing phalloplasty post-penectomy for penile cancer.
- Studies reporting surgical techniques, functional outcomes, and complications.
- Articles offering insights into patient counselling or perioperative management.
- Studies discussing phalloplasty in any cohort of patients where data on post-penectomy for penile cancer were extractable.

Exclusion criteria were:

- Studies focusing solely on gender-affirming surgeries or phalloplasty unrelated to oncological indications.
- Literature without clear documentation of outcomes or techniques.

## Results

**Fig. 1:**
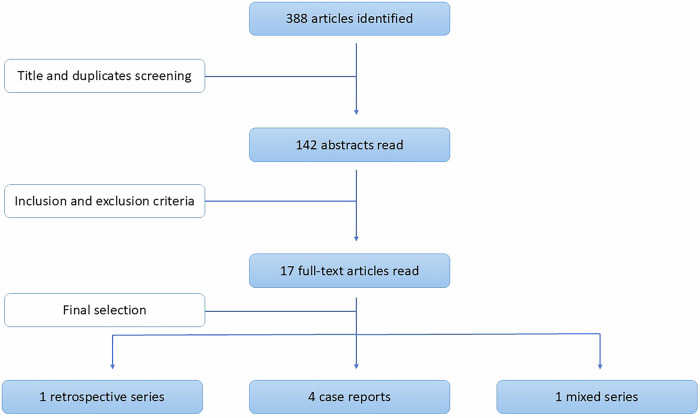
Flowchart diagram. The figure illustrates the selection process.

Figure 1 shows the flowchart diagram and selection process. A total of 388 studies were identified through the MEDLINE and Embase database searches. After the initial screening by title, 142 articles were selected for abstract review. Seventeen full-text articles were assessed, and finally, six articles were included in this narrative review: 1 retrospective case series, 4 case reports, and 1 mixed series with extractable data on penile cancer patients [12, 15–19]. We reviewed the references of the full-text articles but did not find any other relevant studies. The available evidence is scarce and of very low quality, with no prospective studies or randomised trials identified.

Overall, data on 48 individuals were reported in studies published between 1999 and 2021, with follow-up (FU) ranging from 1 to 150 months (overall mean of 77.4 months, Table 1). FU is generally short, except in the study by Falcone et al., which reported outcomes with a median FU of 116 months [19]. The age at the time of surgery ranged from 16 to 63 years, and in all cases, the surgical indication was reconstruction following treatment for penile cancer. Complications are reported in all studies; however, incidence can only be extrapolated from the 2 published case series [15, 19]. Functional outcomes and complications are discussed further in the next section. Table 1 summarises the outcomes reported in the included studies.

**Table 1:** summarises the available evidence and outcomes of phalloplasty after penectomy for penile cancer.

Satisfied (%)	100	100	100	100	100	80
**Able to reach orgasm (%)**	100	NR	100	NR	100	76
**Sensate phallus (%)**	100	NR	100	87	100	80
**Able to void standing (%)**	100	100	100	100	100	
**FU duration (median, m)**	48	12	12	20	84	116
**Population age (mean, y)**	51	16	43	44	16	47
**Recruitment years**	1998	2006	2008	1998-2008	2013	2001-2018
**Number of patients**	1	1	2	15	1	28
**Author**	Sasaki et al.	Hoebeke et al.	Lee et al.	Garaffa et al.	Akino et al.	Falcone et al.

*FU* follow-up, *y* years, *m* months.

## Discussion

### Surgical techniques

**Fig. 2:**
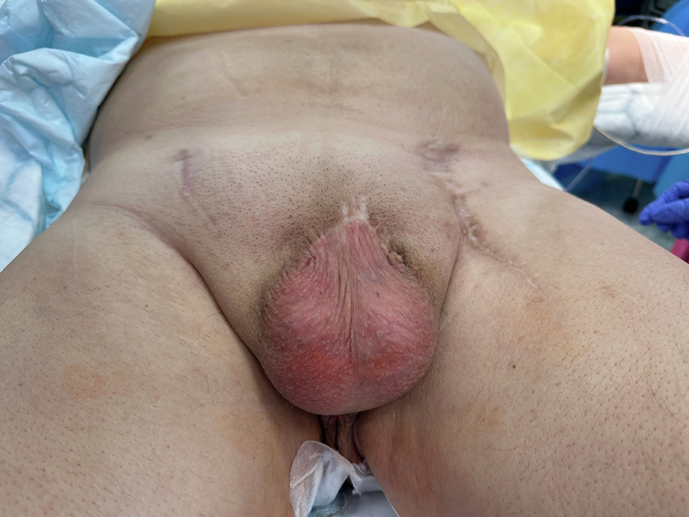
Penile amputation. The figures shows the outcomes of penile amputation with perineal urethrostomy.

Phalloplasty following penectomy for penile cancer necessitates advanced surgical approaches to reconstruct a neophallus capable of providing both functional and aesthetic restoration (Fig. 2). The challenges are heightened by anatomical and vascular compromise caused by oncological surgery, requiring tailored techniques for each patient.

**Fig. 3:**
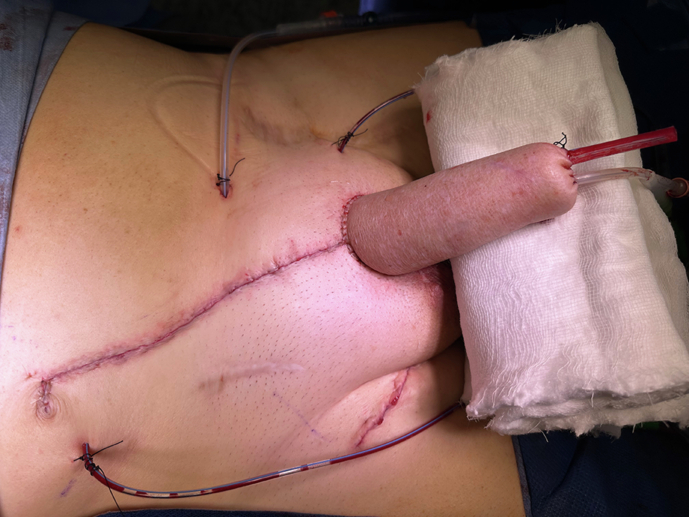
Outcome fo first stage phalloplasty. The patient has a drain inside the phallus to prevent hematoma, a suprapubic catheter, and a transurethral catheter.

**Fig. 4:**
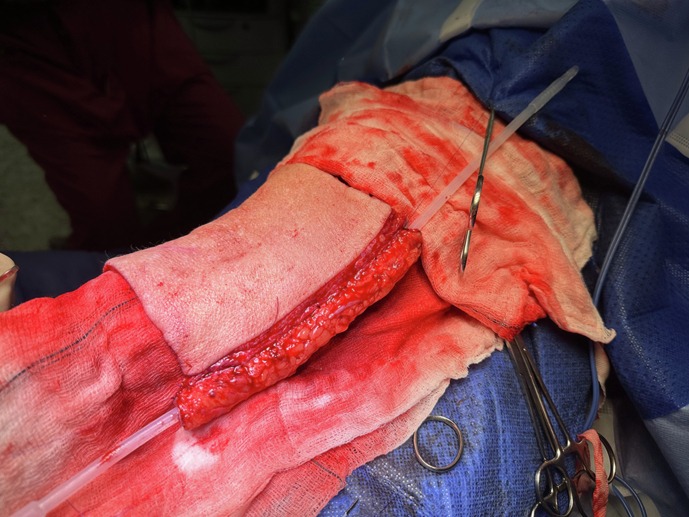
Urethra construction. Urethral tabularisation around a 16ch catheter following the harvest of the radial forearm flap at the time of the first stage.

**Fig. 5:**
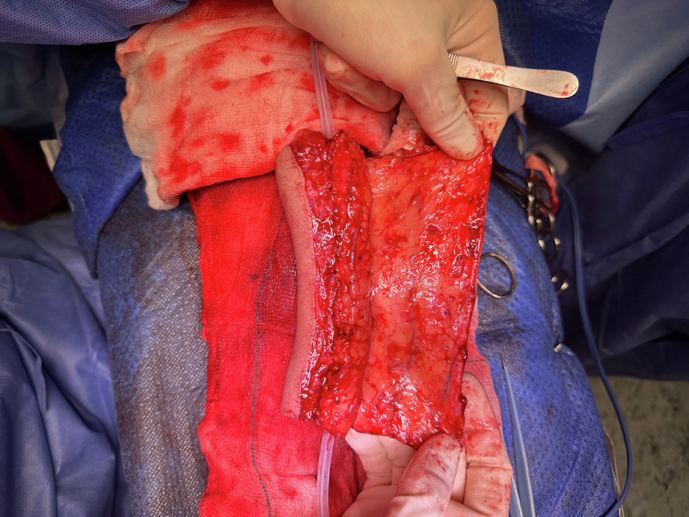
Phallus construction. Construction of the phallus with integrated urethra by tubularising the phallus around the urethra. This technique is also referred to as “tube-in-tube”.

**Fig. 6:**
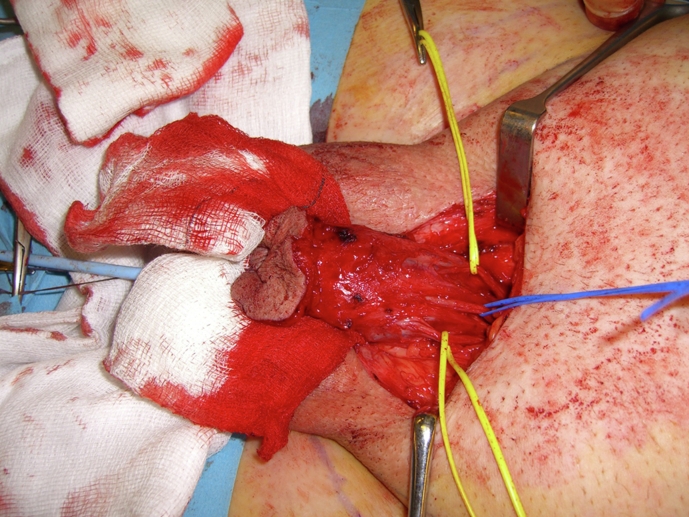
Penile neurovascular bundle. The figure illustrates the 2 dorsal penile nerves (marked by the yellow sloops) and the superficial dorsal penile vein (blue sloop). One of the nerves and the vein can be used for microsurgical anastomoses following penile amputation for cancer when viable.

Key to this is urethral reconstruction and the current gold standard requires an integrated urethra as part of a free or pedicled tissue flap. Among these, the RAP is preferred due to its reliability and versatility (Fig. 3) [15, 20]. Originating from the advancements by Chang and Hwang, the RAP technique involves raising a fasciocutaneous tissue flap from the forearm based on the radial artery pedicle [21]. This method allows for urethral integration using the “tube-within-a-tube” concept, which has a lower risk for subsequent complications when compared to prelaminated techniques (Figs. 4 and 5) [22]. The radial artery supplies arterial blood flow, while venous drainage is established through the vena comitantes, cephalic and basilic veins. Sensory recovery is achieved by neurorraphy between the lateral antebrachial cutaneous nerves with the dorsal penile and ilio-inguinal nerves, resulting in a high rate of tactile and erogenous sensation (Fig. 6) [15, 18, 20, 23, 24]. However, the technique is not without limitations, as significant donor site morbidity, including visible scarring, reduced sensation, and potential for contractures, remains a concern [11, 15, 20, 25]. Additionally, the need for advanced microsurgical expertise and the mismatch in skin colour between the forearm and recipient site add to the disadvantages of using this flap.

An alternative to the RAP is the ALTP, which has gained popularity among patients who prefer a more concealed donor site [12]. Unlike the RAP, the ALT flap is often pedicled, eliminating the need for microvascular anastomosis and thereby reducing the risk of thrombosis in these vessels [26–29]. However, if the vascular pedicle is too short to allow transposition of the phallus onto the pubis, it must be transected and anastomosed similarly to RAP [30]. This technique provides sufficient bulk for neophallus construction, making it particularly useful for patients with limited subcutaneous fat or who prefer a more girthy phallus [31, 32]. However, the technique is not without challenges. The subcutaneous thickness of the ALT flap can complicate tabularisation, especially in patients with higher fat deposition, and the variable vascular anatomy can make flap harvest technically demanding [26]. Sensory recovery, while possible through neurorraphy between the lateral femoral cutaneous and dorsal penile nerves, tends to be less predictable compared to the RAP [20, 27]. Despite these drawbacks, the ALT flap offers a superior aesthetic outcome due to its better colour match and reduced risk of donor site morbidity [28, 32].

Other techniques such as the musculocutaneous latissimus dorsi (MLD) flap and abdominal flap have more limited applications. The MLD flap, frequently employed in gender-affirming surgery, provides a substancial neophallus but suffers from poor tactile sensation and the need for staged prelaminated urethral reconstruction [33, 34]. Abdominal flaps, while technically less demanding, are now rarely used owing to their poor cosmetic outcomes and limited functional benefits [22, 35]. They may be considered in select cases where other options are contraindicated. Both these flaps have not been reported in men following penectomy.

Phalloplasty in cisgender males following penectomy presents unique surgical challenges compared to gender-affirming surgeries. Scar tissue from previous surgery often necessitates alternative venous and neural anastomoses due to the loss of structures such as the long saphenous vein from the primary oncological surgery [8]. These risks are due to the poor vascularisation at the recipient site, making integrated urethral designs technically demanding. Selecting an appropriate donor site involves balancing cosmetic outcomes, vascular reliability, and patient preferences. These factors complicates surgical planning in a way similar to penile reconstruction in patients with exstrophy-epispadias complex [36]. Regarding the timing of surgery, a phalloplasty can be offered after a minimum of 1 year of recurrence-free survival [16]. Although immediate reconstruction with phalloplasty is technically feasible, subsequent tumour recurrence has been reported [17].

### Functional results

Most advancements in phalloplasty have been driven by techniques initially developed for masculinising gender-affirming surgery [21]. Consequently, only limited evidence exists for this specific population, including a single retrospective study involving 15 patients [15] and several case reports and mixed series [12, 16–19]. Table 1 summarises the available outcomes data.

Nevertheless, functional and cosmetic results of RAP were promising, with a median FU of 20 months (range 1–68) [15]. Most patients expressed satisfaction with the appearance and dimensions of the neophallus. Sensory recovery was reported in 87% of cases, while 5 of 6 men with a penile prosthesis (also called erectile device) successfully engaged in sexual activity.

The insertion of erectile devices enhanced key domains such as erectile and orgasmic function, intercourse satisfaction, and overall sexual well-being, as assessed by the International Index of Erectile Function questionnaire [37, 38]. Despite these findings, no significant improvements in overall quality of life were observed following device implantation. It is important to note that these studies predominantly included patients with congenital conditions like micropenis or exstrophy, making direct comparisons to individuals undergoing reconstruction after penile cancer difficult [37, 38]. The latter group may experience poorer outcomes related to quality of life, health satisfaction, and body image, potentially due to the contrast between their previous normal anatomy and the reconstructed neophallus [1–4].

### Complications

Complications are common, reflecting the complexity of reconstruction in this context. Urethral issues, such as strictures or fistulae, are the most frequent, occurring in nearly half of the cases [39–41]. Infections lead to erectile device explantation in up to 14% of patients, while donor site morbidities included incomplete skin graft take, radial nerve sensory loss, lymphoedema, and contractures, each affecting a minority of cases [39–42]. The risk of complications is heavily influenced by factors such as the flap type and the recipient site’s anatomical characteristics. For instance, in the authors’ experience, urethral complications are more likely if a native, vascularised bulbar urethral segment is unavailable for anastomotic urethroplasty. This variability makes it difficult to draw meaningful comparisons between patient cohorts, such as those undergoing phalloplasty for gender affirmation versus post-penectomy individuals. Although systematic reviews have attempted to compare complication rates between these groups, such analyses must be approached with caution due to significant anatomical and surgical heterogeneity [9, 41]. Nevertheless, some studies suggest that cisgender men may achieve better outcomes than transgender men. In a review by Remington et al., aggregated complication rates were compared between cisgender and transgender men [41]. Urethral and flap complication rates were higher among transgender men (39.4% vs. 24.8% and 10.8% vs. 8.1%, respectively), and functional outcomes were poorer, particularly concerning the ability to void while standing (73% vs. 82.8%) and sexual function (51.1% vs. 71.4%). In contrast, a more recent study by Paganelli et al. found no significant differences in the rates of fistulae and strictures between transgender and cisgender males (71.8% vs. 72.7%) [43]. A further study specifically examined outcomes in cisgender men, offering data relevant to penile cancer patients [19]. In this cohort, 108 cisgender males underwent RAP reconstruction, including 28 patients following penile cancer treatments. Among this subset, urethral complications were reported in 64.3% of cases, and 17.9% experienced partial penile necrosis. Despite these complications, overall satisfaction and the ability to achieve orgasm were high across the entire cohort, reported at 80% and 76%, respectively.

Finally, detailed description of the potential complications in penile cancer patients was reported by Garaffa et al [15]. In their cohort of 15 patients, the authors reported complications affecting both the phallus and the arm. In the phallus, they observed 2 cases of partial skin necrosis, 2 contractures, 3 meatal strictures, 1 anastomotic stricture, 5 urethral fistulae, and 1 prosthetic implant explantation. Regarding the arm, 2 patients experienced incomplete graft take, 2 graft contractures, 2 instances of loss of sensation, and 1 case of persistent hand oedema. Notably, all patients were satisfied with the size and cosmesis of the phallus. Unfortunately, other studies involving cisgender men with different indications for phalloplasty did not report outcomes specific to penile cancer patients and were therefore excluded from this review.

### Senior authors’ experience

One of the foremost challenges lies in addressing the anatomical and vascular disruptions caused by oncological surgery. Scarring at the recipient site frequently complicates venous and neural anastomoses requiring adaptation. For example, surgeons may need to utilise alternate recipient vessels such as the femoral/penile dorsal vein or explore less conventional neural connections, like the iliohypogastric or genital branch of the genitofemoral nerves, to restore sensation [8, 15, 36]. Anastomotic urethroplasty is preferred from a functional perspective but complications such as strictures and fistulas will be higher when compared to a perineal urethrostomy [15]. These risks are higher still if there is compromised vascularity, emphasising the importance of patient-specific surgical strategies. However, patients should be aware that if the quality of the remnant urethra is poor, anastomotic urethroplasty should be delayed, particularly if graft augmentation is required. The authors routinely stage the procedure as this approach facilitates reassessment of urethral quality at the time of tubularisation. It also allows for further adjustments if necessary, as it was shown in the cis male population [44].

The psychosocial complexities of phalloplasty are equally significant. Patients undergoing this procedure often experience profound emotional distress arising from the dual impact of cancer and genital loss [1–4]. Counselling must therefore extend beyond standard preoperative discussions to address issues of identity, intimacy, and long-term adaptation. Engaging a multidisciplinary team, including psychologists, social workers, and sexual health counsellors, is essential [45–48]. Such support is not only crucial for managing expectations but also for facilitating informed decision-making. This holistic approach is difficult to fully implement and is mostly unmet in current practice [48]. In our referral centre, all men requesting phalloplasty are seen and counselled by a dedicated team of psychologists and clinical nurse specialists, in addition to the surgical team. The impact of this intervention and how it will affect outcomes remains to be clarified but addressing the psychological challenges these individuals face should be prioritised. Patients are counselled that while a reconstructed phallus can allow standing micturition and improved self-image, achieving erogenous sensation and erections often requires additional interventions, such as erectile device implantation and can lead to higher rates of subsequent complications [8, 15, 36]. Tailoring counselling to an individual needs is particularly vital, as the goals and expectations of patients may vary widely based on factors such as age, cultural background, and prior sexual activity.

Another challenge lies in the extrapolation of data from the transgender population. Much of the existing literature on phalloplasty outcomes, including data on techniques like the RAP and ALTP, derives from gender-affirming surgery. A previous review demonstrated that only 5 studies focused on phalloplasty following surgery for penile cancer [8]. Also, another systematic review on total penile reconstruction by Yao et al. found that, of the 925 RAP included, 823 were performed because of gender dysphoria, leaving only a minority of the surgeries performed in different populations (such as bladder exstrophy, penile insufficiency, etc) [9]. While these studies offer valuable insights into technical feasibility and complication rates, their applicability to cisgender males following penectomy is limited. For example, transgender patients typically undergo phalloplasty in the absence of scarring or prior surgical disruptions, whereas post-penectomy patients often present with significant anatomical changes. Moreover, the possible presence of the proximal crural and spongiosal bodies may simplify the surgical technique. This disparity highlights the urgent need for dedicated investigation focusing specifically on cisgender males with oncological indications.

Future research must address several critical gaps. Large-scale studies are challenging to conduct in this specific population. Establishing multicentre databases or registries, with the support of specialty societies, could help overcome the challenge of limited patient numbers. Comparative studies evaluating outcomes between cisgender and transgender populations would provide more tailored guidance for surgical planning. Additionally, long-term FU studies are needed to assess the durability of functional and aesthetic results, as well as the psychosocial impact of these procedures. Innovations in tissue engineering, such as bioengineered grafts for urethral reconstruction, also hold promise for reducing complications and improving outcomes. Finally, expanding educational opportunities and mentorship for surgeons through specialised training programs could enhance the quality and consistency of care.

## Conclusion

Phalloplasty for men following penile cancer treatment is feasible, with good reported satisfaction rates despite the significant risks of complications. Various surgical techniques for phalloplasty after penectomy for penile cancer have been described but high-quality, large-scale studies are lacking. Most available data primarily come from transgender populations, limiting the generalisability to cisgender men. It is likely that outcomes in cisgender males tend to be better than in transgender males, with high functional outcomes and satisfaction rates. However, the complexity of the surgical procedures, along with the need for comprehensive counselling and long-term FU, underscores the importance of further research. Future studies should focus on standardising techniques, improving outcomes, and providing robust data to guide clinical practice in this challenging area.
